# High dimensional and high resolution pulse sequences for backbone resonance assignment of intrinsically disordered proteins

**DOI:** 10.1007/s10858-012-9613-x

**Published:** 2012-02-17

**Authors:** Anna Zawadzka-Kazimierczuk, Wiktor Koźmiński, Hana Šanderová, Libor Krásný

**Affiliations:** 1 Faculty of Chemistry, University of Warsaw, Pasteura 1, 02-093 Warsaw, Poland; 2 Laboratory of Molecular Genetics of Bacteria, Department of Bacteriology, Institute of Microbiology, Academy of Sciences of the Czech Republic, Vídeňská 1083, 142 20 Prague, Czech Republic

**Keywords:** Intrinsically disordered proteins, Non-uniform sampling, Backbone assignment, 5D NMR, 6D NMR

## Abstract

Four novel 5D (HACA(N)CONH, HNCOCACB, (HACA)CON(CA)CONH, (H)NCO(NCA)CONH), and one 6D ((H)NCO(N)CACONH) NMR pulse sequences are proposed. The new experiments employ non-uniform sampling that enables achieving high resolution in indirectly detected dimensions. The experiments facilitate resonance assignment of intrinsically disordered proteins. The novel pulse sequences were successfully tested using δ subunit (20 kDa) of *Bacillus subtilis* RNA polymerase that has an 81-amino acid disordered part containing various repetitive sequences.

## Introduction

Intrinsically disordered proteins (IDPs) are a group of macromolecules with peculiar structure, dynamics and interactions (Dyson and Wright 2001; Tompa et al. 2006) that allow them to play a number of important biological functions (Ward et al. 2004; Dyson and Wright 2005; Tompa 2010). Solution state NMR spectroscopic techniques appear ideally suited for the studies of these proteins as IDPs’ fast conformational dynamics results in relatively slow transverse relaxation rates. The problem is that the rapid interconversion rate for the various conformations causes averaging of chemical shifts and very poor peak separation, making resonance assignment difficult, even for relatively small disordered protein fragments. However, there is a promise for solving this obstacle in multidimensional NMR methods utilizing non-uniform sampling of indirectly detected dimensions (Felli and Brutscher 2009; Coggins et al. 2010; Kazimierczuk et al. 2010a), as this sampling facilitates acquisition of high-resolution and high-dimensional spectra.

**Fig. 1:**
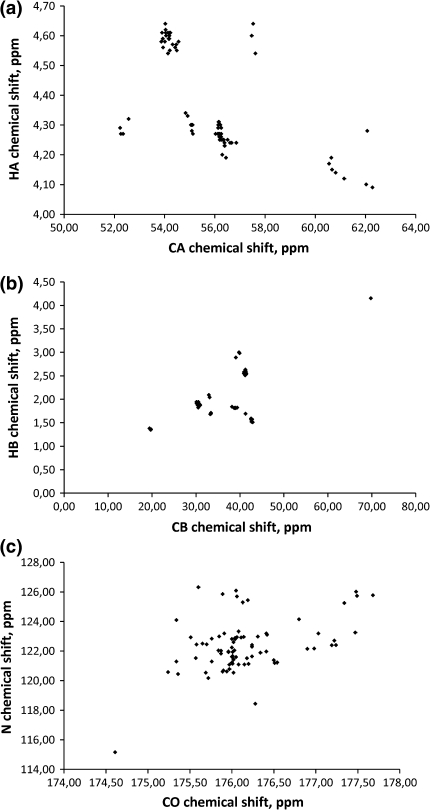
Correlation of HA–CA (**a**), HB–CB (**b**), and N–CO (**c**) chemical shifts for the 81 a.a. unstructured part of the δ subunit of *B. subtilis* RNA polymerase. The ^1^H and ^13^C chemical shifts depend mostly on particular amino acid residue, whereas ^13^CO and ^15^N frequencies are much better resolved and enable identification of backbone connectivity. (BMRB entry 16912)

The established approach for backbone resonance assignment of globular proteins consists of a suite of triple-resonance experiments where sequential connectivities are found using carbonyl ^13^C, aliphatic ^13^C and/or aliphatic ^1^H chemical shifts (Sattler et al. 1999). However, this approach often fails in the case of IDPs because of poor dispersion of side-chain chemical shifts (which depend mostly on the residue identity). In Fig. 1, we show HA-CA, HB-CB and N-CO chemical shift correlations for the 81 a.a. unstructured part of the δ subunit of *B. subtilis* RNA polymerase (Motáčková et al. 2010; Nováček et al. 2011). These graphs clearly show the best dispersion of amide nitrogen and carbonyl carbon chemical shifts. The utility of these nuclei for sequential assignment is additionally supported by their relatively slow (as compared to that of aliphatic protons or aliphatic carbon atoms) transverse relaxation and lack of non-refocused homonuclear couplings, the presence of which may limit resolution even stronger than does the relaxation limit, e.g. in the case of aliphatic carbon atoms.

To date, several strategies have been proposed for effective backbone resonance assignment of IDPs. These strategies include ^13^C detection (Bermel et al. 2006, 2009), automated projection spectroscopy (APSY; Narayanan et al. 2010), HA detection (Mäntylahti et al. 2011), and the sparsely sampled 4D (Wen et al. 2011) and 5D experiments (Motáčková et al. 2010; Nováček et al. 2011). Below, we propose a set of new pulse sequences that feature high resolution and high dimensionality resulting from the use of sparse random sampling in the indirectly detected dimensions. The novel experiments, which require (because of multiple coherence transfer steps involved) a slow transverse relaxation, were designed for IDPs and are superior in terms of peak resolution and the easiness of resonance assignment of the proteins. The pulse sequences were tested using the 20 kDa δ subunit of *B. subtilis* RNA polymerase. Having an 81 a.a. unstructured part with various repetitive sequences, this macromolecule is an excellent example of an IDP whose resonance assignment is extremely difficult using conventional methods.

## Methods

The uniformly ^13^C,^15^N-labeled sample of *B. subtilis* RNA polymerase δ subunit was prepared as described previously (Motáčková et al. 2010). All spectra were acquired in a 0.7 mM protein solution sample on a Varian NMR System 700 spectrometer equipped with a Performa XYZ PFG unit, using the standard 5 mm ^1^H-^13^C-^15^N triple-resonance probehead. High-power ^1^H, ^13^C and ^15^N π/2 pulses of 5.9, 13.5 and 31.0 μs, respectively, were used. Selective CA and CO pulses were realized as phase-modulated (for off-resonance excitation or inversion) *sinc* shapes, with B_1_ field strength adjusted to have a minimal effect on CO and CA, respectively. In all cases, four scans per each data set were acquired with acquisition time of 85 ms and relaxation delay of 1.2 s. For processing of directly detected dimension, cosine square weighting function was used prior to Fourier transform with zero-filling to 2,048 complex points. The experiments were performed using random off-grid Poisson disk sampling with sampling density set according to a Gaussian distribution (σ = 0.5) with regard to maximum evolution time (Kazimierczuk et al. 2008). No apodization was applied in indirect dimensions. The number of complex points M_i_ in the frequency domain of *i*th indirectly detected dimension was set as M_i_ ≥ 3 × sw_i_ × t_i_^max^. The sparse multidimensional Fourier transform (SMFT) procedure (Kazimierczuk et al. 2009), with ‘fixed’ frequencies derived from 3D HNCO and 4D HNCOCA peak list, was used to obtain *F*_1_/*F*_2_ cross-sections in 5D and 6D experiments, respectively. The remaining relevant experimental parameters are shown in Table 1.

**Table 1:** Maximum evolution times (*t* ^max^, ms) and spectral width (sw, kHz) used for acquisition of spectra for *B. subtilis* RNA polymerase δ subunit

	5D HACA(N)CONH	5D HNCOCACB	5D (H)NCO(NCA)CONH	6D (H)NCO(N)CACONH	5D (HACA)CON(CA)CONH
Number of points	1,000	1,300	1,570	1,000	770
Experiment duration (h)	23.0	29.5	36.0	45.5	17.5
sw_1_	4	14	2.8	2.8	3.8
sw_2_	6.2	14	3	3	3
sw_3_	3	3	3	6.2	3
sw_4_	2.8	2.8	2.8	3	3.8
sw_5_	n.a.	n.a.	n.a.	2.8	n.a.
*t*_1_^max^	20	10	50	50	50
*t*_2_^max^	10	10	45	45	45
*t*_3_^max^	45	45	45	28.6	45
*t*_4_^max^	75	75	75	45	75
*t*_5_^max^	n.a.	n.a.	n.a.	75	n.a.
Sampling density versus conventional	7.11 × 10^−6^	2.34 × 10^−6^	2.93 × 10^−6^	1.05 × 10^−8^	7.80 × 10^−7^

The pulse sequences were written using own-developed programming library. The resulting spectra were analyzed using the SPARKY software (Goddard and Kneller 2002). The pulse sequence code for Agilent spectrometers as well as the SMFT software used for data processing are available from the authors upon request.

## Results and discussion

**Fig. 2:**
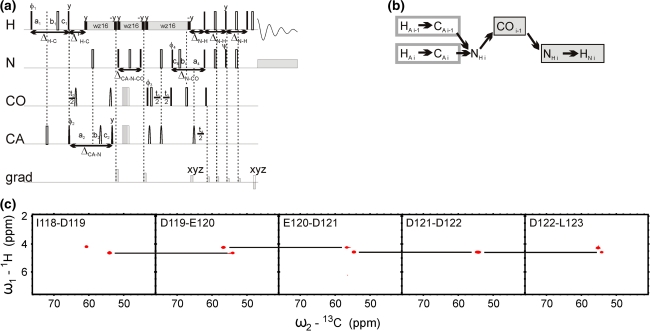
5D HACA(N)CONH technique. (**a**) Pulse sequence, ^1^H, ^13^CA, and ^15^N evolution is in semi-constant-time mode: a_i_ = (*t*_i_ + Δ)/2, *b*_i_ = *t*_i_(1−Δ/*t*_i_^max^)/2, *c*_i_ = Δ(1−*t*_i_/*t*_i_^max^)/2 (where Δ stands for coherence transfer delays listed below, *t*_i_ is the evolution time in *i*th dimension, and *t*_i_^max^ is maximal length of the evolution time delay). Delays were set as follows: Δ’_H-C_ = 2.6 ms, Δ_CA-N_ = 28.0 ms, Δ_CA-N-CO_ = 28.0 ms, Δ_N-CO_ = 28.0 ms, and Δ_N-H_ = 5.4 ms. The four-step phase cycle was used: ϕ_1_ = x, −x, ϕ_2_ = 2x, 2(−x) and Rec = ϕ_1_ + ϕ_2_. Simultaneous inversion of CA and CO spins was achieved using 6-element composite pulse (Shaka 1985). The coherence selection gradients (*marked xyz*) were applied at the magic angle. The phase ψ was inverted simultaneously with the last gradient pulse. (**b**) Coherence transfer in the peptide chain. H^N^, N, and CO frequencies (*filled rectangles*) are ‘fixed’ for Fourier transform. Frames for HA and CA indicate the dimensions of 2D cross-sections obtained by SMFT procedure. (**c**) 2D spectral planes for the δ subunit of *B. subtilis* RNA polymerase, which were obtained by SMFT procedure performed on the 5D randomly sampled signal (Poisson disk sampling) with ‘fixed’ frequencies obtained from 3D HNCO peak list. Each cross-section contains two cross-peaks: for’fixed’ H_i_^N^, N_i_ and CO_i−1_, the peaks correspond to HA_i_–CA_i_ and HA_i−1_–CA_i−1_ correlations

**Fig. 3:**
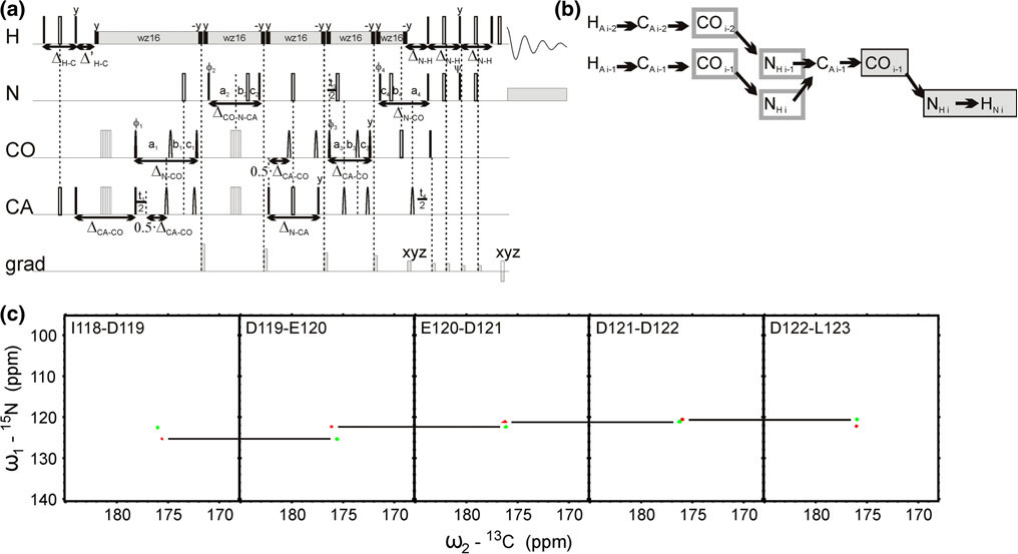
5D (HACA)CON(CA)CONH technique. (**a**) Pulse sequence, ^15^N (in *t*_2_ and *t*_4_) and ^13^CO (in *t*_1_ and *t*_3_) evolution is in semi-constant-time mode: a_i_ = (*t*_i_ + Δ)/2, *b*_i_ = *t*_i_(1−Δ/*t*_i_^max^)/2, *c*_i_ = Δ(1−*t*_i_/*t*_i_^max^)/2 (where Δ stands for coherence transfer delays listed below, *t*_i_ is the evolution time in *i*th dimension, and *t*_i_^max^ is maximal length of the evolution time delay). Delays were set as follows: Δ_H-C_ = 3.7 ms, Δ’_H-C_ = 2.6 ms, Δ_CA-CO_ = 6.8 ms, Δ_CA-N_ = 28.0 ms, Δ_CA-N-CO_ = 28.0 ms, Δ_N-CO_ = 28.0 ms, Δ_N-H_ = 5.4 ms. The four-step phase cycle was used: ϕ_1_ = x, −x, ϕ_2_ = 2x, 2(−x) and Rec = ϕ_1_ + ϕ_2_. Simultaneous inversion of CA and CO spins was achieved using 6-element composite pulse (Shaka 1985). The coherence selection gradients (*marked by xyz*) were applied at the magic angle. The phase ψ was inverted simultaneously with the last gradient pulse. (**b**) Coherence transfer in the peptide chain. H^N^, N, and CO frequencies (*filled rectangles*) are ‘fixed’ for Fourier transform. Frames for N and CO indicate the dimensions of 2D cross-sections obtained by SMFT procedure. (**c**) 2D spectral planes for the δ subunit of *B. subtilis* RNA polymerase, which were obtained by SMFT procedure performed on the 5D randomly sampled signal (Poisson disk sampling) with ‘fixed’ frequencies obtained from 3D HNCO peak list. Each cross-section contains two cross-peaks: for ‘fixed’ H_i_^N^, N_i_ and CO_i−1_ the peaks correspond to N_i_-CO_i−1_ and N_i−1_-CO_i−2_ correlations

The first two pulse sequences (5D HACA(N)CONH and 5D (HACA)CON(CA)CONH) are depicted schematically in Figs. 2a and 3a, respectively, and the corresponding coherence transfer pathways are given in Figs. 2b and 3b. Both experiments employ equilibrium magnetization of HA protons. This allows identifying certain chemical shifts of the proline residues whose successors’ amide protons are detected. In both experiments, the effective separation of *F*_1_/*F*_2_ cross-sections is obtained due to the good peak separation in CO–N subspectra. In the HACA(N)CONH experiment, the sequential connectivities may be obtained from ^1^HA and ^13^CA chemical shifts, which approach usually fails in the case of IDPs due to poor peak separation. On the other hand, the (HACA)CON(CA)CONH spectra allow, at the expense of additional coherence transfer steps, finding of connectivities with the use ^13^CO and ^15^N frequencies that are more uniformly distributed over the entire spectral band and therefore are more suitable for the studies of IDPs. This experiment requires extension of the spectral width in the first two dimensions to accommodate correlations of proline residues. In Figs. 2c and 3c we show an example of 2D cross-sections for the I118-L123 fragment of the disordered part of δ subunit of *B. subtilis* RNA polymerase.

**Fig. 4:**
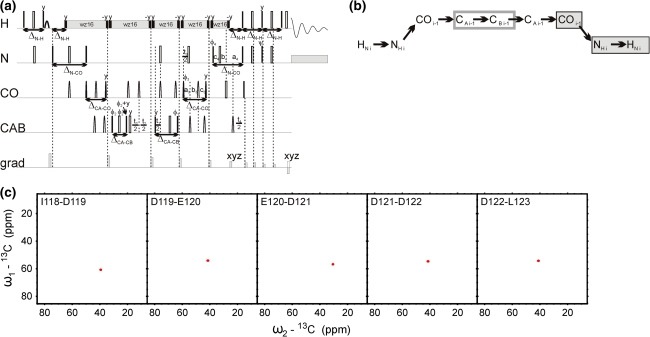
5D HNCOCACB technique. (**a**) Pulse sequence, ^13^CO and ^15^N evolution is in semi-constant-time mode: a_i_ = (*t*_i_ + Δ)/2, *b*_i_ = *t*_i_(1−Δ/*t*_i_^max^)/2, *c*_i_ = Δ(1−*t*_i_/*t*_i_^max^)/2 (where Δ stands for listed below coherence transfer delays, *t*_i_ is the evolution time in *i*th dimension and *t*_i_^max^ is maximal length of the evolution time delay). ^13^CA chemical shift evolution is in constant-time mode. Delays were set as follows: Δ_N-H_ = 5.4 ms, Δ_N-CO_ = 28.0 ms, Δ_CA-CO_ = 9.1 ms, and Δ_CACB_ = 14.3 ms. The four-step phase cycle was used: ϕ_1_ = x, −x, ϕ_2_ = 2x, 2(−x) and Rec = ϕ_1_ + ϕ_2_. The coherence selection gradients (*marked by xyz*) were applied at the magic angle. The phase ψ was inverted simultaneously with the last gradient pulse. (**b**) Coherence transfer in the peptide chain. H^N^, N, and CO frequencies (*filled rectangles*) are ‘fixed’ for Fourier transform. Frames for CA and CB indicate the dimensions of 2D cross-sections obtained by SMFT procedure. (**c**) 2D spectral planes for the δ subunit of *B. subtilis* RNA polymerase, which were obtained by SMFT procedure performed on the 5D randomly sampled signal (Poisson disk sampling) with ‘fixed’ frequencies obtained from 3D HNCO peak list. Each cross-section contains one cross-peak: for ‘fixed’ H_i_^N^, N_i_ and CO_i−1_ the peak corresponds to CA_i−1_-CB_i−1_ correlation

The out-and-back 5D HNCOCACB experiment shown in Fig. 4 correlates ^1^H_i_^N^, ^15^N_i_, ^13^CO_i−1_ with ^13^CA _i−1_ and ^13^CB _i−1_ chemical shifts. Contrary to the established CBCANH, CBCA(CO)NH and HNCACB experiments (Grzesiek and Bax 1992a, 1992b, 1993; Wittekind and Müller 1993), the CA and CB evolutions are performed in separate dimensions. This allows to increase the CA-CB coupling evolution delay to 0.5/J_CACB_ and therefore, to double (providing one ignores the relaxation) the sensitivity, which in the case of IDPs compensates for the extended pulse sequence. Although this sequence does not provide sequential connectivities, it allows to assign CA and CB chemical shifts and to identify a.a. residues by comparing the respective chemical shifts with typical values for each amino acid.

**Fig. 5:**
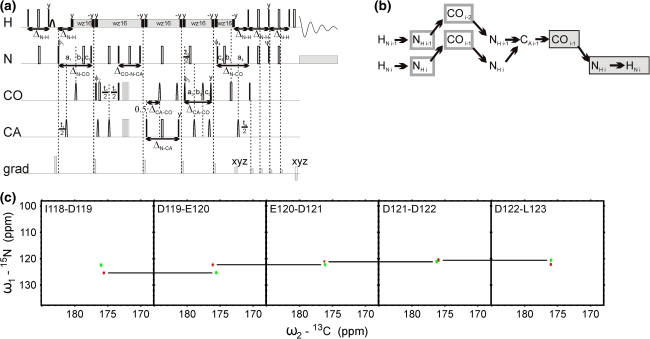
5D (H)NCO(NCA)CONH technique. (**a**) Pulse sequence, ^15^N (in *t*_1_ and *t*_4_) and ^13^CO (in *t*_3_) evolution is in semi-constant-time mode: a_i_ = (*t*_i_ + Δ)/2, *b*_i_ = *t*_i_(1−Δ/*t*_i_^max^)/2, *c*_i_ = Δ(1−*t*_i_/*t*_i_^max^)/2 (where Δ stands for listed below coherence transfer delays, *t*_i_ is the evolution time in *i*th dimension and *t*_i_^max^ is maximal length of the evolution time delay). Delays were set as follows: Δ_N-H_ = 5.4 ms, Δ_N-CO_ = 28.0 ms, Δ_CO–N-CA_ = 28.0 ms, Δ_N-CA_ = 28.6 ms, and Δ_CA-CO_ = 9.1 ms. The four-step phase cycle was used: ϕ_1_ = x, −x, ϕ_2_ = 2x, 2(−x) and Rec = ϕ_1_ + ϕ_2_. Simultaneous inversion of CA and CO spins was achieved using 6-element composite pulse (Shaka 1985). The coherence selection gradients (*marked by xyz*) were applied at the magic angle. The phase ψ was inverted simultaneously with the last gradient pulse. (**b**) Coherence transfer in the peptide chain. H^N^, N, and CO frequencies (*filled rectangles*) are ‘fixed’ for Fourier transform. Frames for N and CO indicate the dimensions of 2D cross-sections obtained by SMFT procedure. (**c**) 2D spectral planes for the δ subunit of *B. subtilis* RNA polymerase, which were obtained by SMFT procedure performed on the 5D randomly sampled signal (Poisson disk sampling) with ‘fixed’ frequencies obtained from 3D HNCO peak list. Each cross-section contains two cross-peaks: for ‘fixed’ H_i_^N^, N_i_ and CO_i−1_ the peaks correspond to N_i_-CO_i−1_ and N_i−1_-CO_i−2_ correlations

The 5D (H)NCO(NCA)CONH experiment is schematically depicted in Fig. 5a together with the scheme of coherence transfer pathway in protein backbone 5b. In this case the magnetization of amide proton’s origin is transferred through amide nitrogen and carbonyl carbon nuclei back to nitrogen and to two different CA nuclei, then via the respective CO nuclei to the corresponding coupled NH pairs. In this case, again, ^13^CO and ^15^N chemical shifts enable peak resolution in the ‘fixed’ dimensions *F*_3_ and *F*_4_, and establishing sequential connectivities from ^13^CO and ^15^N in dimensions *F*_1_ and *F*_2_.

**Fig. 6:**
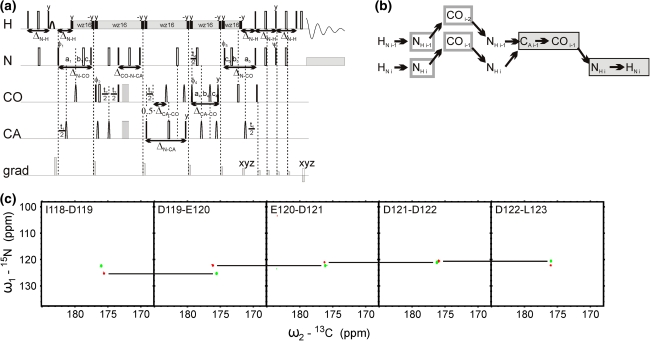
6D (H)NCO(N)CACONH technique. (**a**) Pulse sequence, ^15^N (in *t*_1_ and *t*_5_) and ^13^CO (in *t*_4_) evolution is in semi-constant-time mode: a_i_ = (*t*_i_ + Δ)/2, *b*_i_ = *t*_i_(1−Δ/*t*_i_^max^)/2, *c*_i_ = Δ(1−*t*_i_/*t*_i_^max^)/2 (where Δ stands for listed below coherence transfer delays, *t*_i_ is the evolution time in *i*th dimension and *t*_i_^max^ is maximal length of the evolution time delay). CA evolution (in *t*_3_) is in constant-time mode. Delays were set as follows: Δ_N-H_ = 5.4 ms, Δ_N-CO_ = 28.0 ms, Δ_CO-N-CA_ = 28.0 ms, Δ_N-CA_ = 28.6 ms, and Δ_CA-CO_ = 9.1 ms. The four-step phase cycle was used: ϕ_1_ = x, −x, ϕ_2_ = 2x, 2(−x) and Rec = ϕ_1_ + ϕ_2_. Simultaneous inversion of CA and CO spins was achieved using 6-element composite pulse (Shaka 1985). The coherence selection gradients (*marked by xyz*) were applied at the magic angle. The phase ψ was inverted simultaneously with the last gradient pulse. (**b**) Coherence transfer in the peptide chain. H^N^, N, CO and CA frequencies (*filled rectangles*) are ‘fixed’ for Fourier transform. Frames for N and CO indicate the dimensions of 2D cross-sections obtained by SMFT procedure. (**c**) 2D spectral planes for the δ subunit of *B. subtilis* RNA polymerase, which were obtained by SMFT procedure performed on the 6D randomly sampled signal (Poisson disk sampling) with ‘fixed’ frequencies obtained from 4D HNCOCA peak list. Each plane contains two cross-peaks: for ‘fixed’ H_i_^N^, N_i_, CO_i−1_ and CA_i−1_ the peaks correspond to N_i_-CO_i−1_ and N_i−1_-CO_i−2_ correlations

**Fig. 7:**
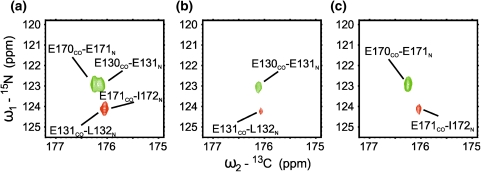
Comparison of 2D N-CO cross-sections from 5D (H)NCO(NCA)CONH (**a**) and 6D (H)NCO(N)CACONH (**b**, **c**) experiments. In 5D experiment (**a**) the two pairs of correlation peaks overlap (E130/E131 with E170/E171, and E131/L132 with E171/I172) due to similarity of chemical shifts for all ‘fixed’ dimensions: H_L132_^N^ and H_I172_^N^, N_L132_ and N_I172_, CO_E131_ and CO_E171_. The additional CA dimension in 6D experiment (**b**, **c**) enabled to differentiate and assign all peaks

The 6D (H)NCO(N)CACONH pulse sequence is shown in Fig. 6. This sequence was obtained from the aforementioned 5D variant by introducing constant time evolution of CA frequencies, i.e. with no increase in overall sequence duration. The extra resolution gain resulting from the increased dimensionality may be crucial for IDPs’ spectra that feature high chemical shift degeneracy. Such an example is given in Fig. 7, where the additional dimension enables to resolve peaks that still overlap in 5D spectra. In this case, however, application of SMFT procedure requires the knowledge of CA_i−1_ chemical shifts. These shifts can be obtained using 5D HNCOCACB, 5D HabCabCONH (Kazimierczuk et al. 2010), or 4D HNCOCA (Zawadzka-Kazimierczuk et al. 2010) experiments.

The experiments shown in Figs. 5 and 6 are conceptually similar to the (HACA)CON(CA)CONH experiment shown in Fig. 3, but the (HACA) fragment is replaced by (H)N. This modification enables application of band-selective excitation short-transient (BEST) approach that is aimed at the acceleration of acquisition (Schanda et al. 2006; Lescop et al. 2007), but does not allow to find the resonances of proline residues. Notably, the (H)NCO(NCA)CONH sequence has a sensitivity advantage over the (HACA)CON(CA)CONH sequence as the CA → CO coherence transfer in the latter is attenuated due to the presence of a concurrent ^1^J_CACB_ coupling. The respective amplitude transfer functions at this point (i.e. before the first CO evolution period) are shown in (1) and (2) below:

1 $$ {\text{I}}({\text{HN}}) = \sin^{2} (\pi {\text{J}}_{\text{NH}} \Updelta_{\text{NH}} )\sin (\pi {\text{J}}_{\text{NCO}} \Updelta_{\text{NCO}} )\exp ( - \Updelta_{\text{NH}} /{\text{T}}_{{2{\text{HN}}}} - \Updelta_{\text{NCO}} /{\text{T}}_{{ 2 {\text{N}}}} ) $$

2 $$ {\text{I(HACA)}} = \sin (\pi {\text{J}}_{\text{HC}} \Updelta_{\text{HC}} )\sin \left( {\pi {\text{J}}_{\text{HC}} \Updelta_{\text{HC}}^{\prime } } \right)\cos^{{{\text{n}} - 1}} \left( {\pi {\text{J}}_{\text{HC}} \Updelta_{\text{HC}}^{\prime } } \right)\sin (\pi {\text{J}}_{\text{CACO}} \Updelta_{\text{CACO}} )\cos (\pi {\text{J}}_{\text{CACB}} \Updelta_{\text{CACO}} ) \, \exp ( - \Updelta_{\text{HC}} /{\text{T}}_{{2{\text{HA}}}} - \Updelta_{\text{CACO}} /{\text{T}}_{{2{\text{CA}}}} ) $$

where n is the number of HA protons (for Gly: n = 2).

We have set Δ_CACO_ = 6.8 ms to compromise between J-couplings and relaxation. The same choice was made by Mäntylahti et al. (2011) for other HA-excited experiments and is usually employed in HSQC type HN(CA)CO experiments (for references see Sattler et al. 1999). Assuming T_2HN_ = 50 ms, T_2N_ = 50 ms, T_2HA_, T_2CA_ = 20 ms, n = 1, and delay times as given in figure captions, we obtain 0.50 and 0.36 for I(HN) and I(HACA), respectively. Setting Δ_CACO_ at 28.5 ms, which is close to 1/J_CACB_, with evolution of J_CACO_ extended to 9.1 ms, further reduces I(HACA) to 0.18. Using the relaxation times T_2HN_ = 80 ms, T_2N_ = 100 ms, T_2HA_ = 40 ms and T_2CA_ = 50 ms, which seem likely for IDPs (based on our experience), and Δ_CACO_ = 6.8 ms, one obtains I(HN) = 0.68 and I(HACA) = 0.49, whereas for Δ_CACO_ of 28.5 ms I(HACA) = 0.47. Therefore, the latter option seems impractical, especially that the relaxation rates and coupling constants may not be uniform in the entire molecule.

Despite long duration of the proposed pulse sequences and high sparsity of the sampling schedules employed we have found all expected peaks for the disordered fragment of *B. subtilis* RNA polymerase δ subunit. We have not found any false peaks, i.e. all resonances found were unambiguously assigned in a sequential manner.

**Fig. 8:**
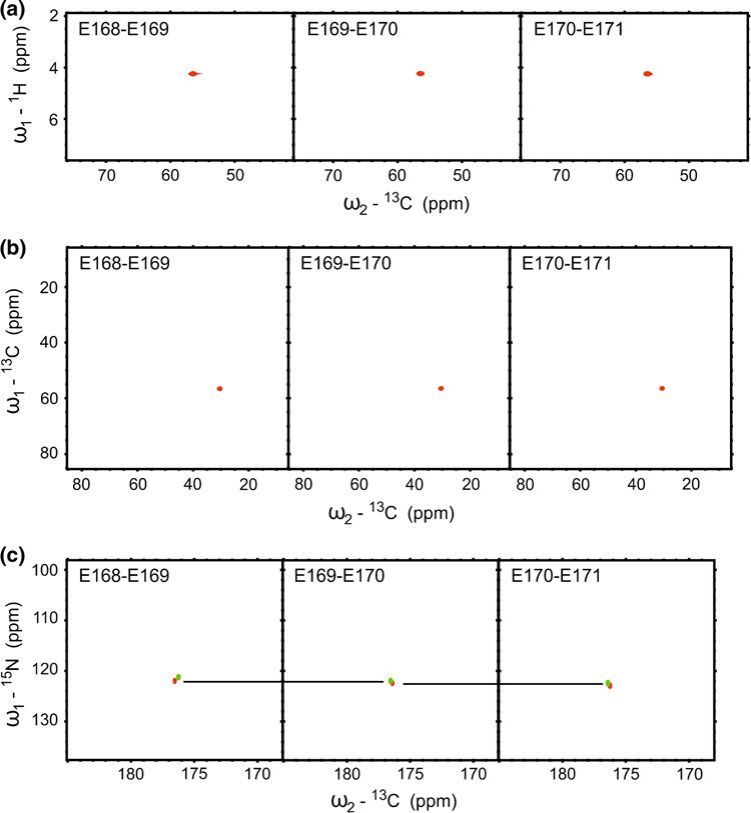
*F*_1_–*F*_2_ 2D cross-sections of HACA(N)CONH (**a**), HNCOCACB (**b**), and (H)NCO(NCA)CONH (**c**) 5D experiments, acquired for the δ subunit of *B. subtilis* RNA polymerase, show E168-E171correlations. The two intra- and inter-residual HA-CA correlation peaks are not resolved in (**a**); CA-CB correlation peaks in (**b**) have identical coordinates, however, the resolved pairs of N-CO peaks shown in (**c**) enable unambiguous sequential assignment

In Fig. 8, non-specificity of aliphatic ^1^H and ^13^C chemical shifts is demonstrated using E168-E171 correlations for δ subunit of RNA polymerase from *B. subtilis*. It is shown that in the repeated glutamic acid fragment the aliphatic chemical shifts do not differ sufficiently for the sequential assignment, while amide nitrogen and carbonyl carbon chemical shifts enable unambiguous assignment.

## Conclusions

Random sampling and SMFT processing allow developing novel NMR experiments of high dimensionality and high resolution that would not be feasible using conventional sampling. The new experiments enable simple and unambiguous backbone assignment of IDPs. Importantly, not all of the presented techniques must be used to obtain complete sequential assignment. One can combine various experiments (also from among those published before) to construct an optimal set for a given protein. The use of the techniques we present in this paper provides sequential connectivities via ^13^CO and ^15^N chemical shifts and enables more straightforward sequential assignment than the 5D experiments published previously. Moreover, the separation of individual spin systems on 2D cross-sections could be very useful for a possible automatic assignment algorithm, which would allow fast and simple resonance assignment, also for large IDPs.
